# Regulation of RHBDD1 in the invasion of esophageal cancer cells via ELK3/Wnt/β-catenin signaling pathway

**DOI:** 10.3389/fbioe.2025.1604859

**Published:** 2025-07-25

**Authors:** Bing Xu, Hui Chen, Xinchen Sun, Hongyan Cheng, Kavimbi Chipusu, Wei Hua, Tingting Chen

**Affiliations:** ^**1**^Department of Oncology, Northern Jiangsu People’s Hospital, Yangzhou, China; ^**2**^Department of Radiation Oncology, The First Affiliated Hospital of Nanjing Medical University, Nanjing, China; ^**3**^Department of Synthetic Internal Medicine, The First Affiliated Hospital of Nanjing Medical University, Nanjing, China; ^4^Department of Mechanical Engineering, Division of Biomedical Engineering, University of Saskatchewan, Saskatoon, Canada

**Keywords:** esophageal squamous cell carcinoma, RHBDD1, Wnt/ β-catenin, invasion, migration

## Abstract

**Objective:**

Esophageal cancer (EC) is one of the most common cancers worldwide. The prognosis for patients with the same stage of EC can vary substantially. Recurrence and metastasis after treatment are still important reasons for poor prognosis of esophageal cancer patients. Rhomboid domain containing 1 (RHBDD1) has been reported to play an important role in the development and progression of various cancers, but its role in esophageal malignancy is poorly understood, and this paper aims to explore the role of RHBDD in esophageal squamous cell carcinoma.

**Methodology:**

This study employed *in vitro* and *in vivo* approaches to investigate molecular mechanisms in ESCC. ECA109 cells were cultured in RPMI 1640 with 10% FBS under 5% CO_2_ at 37°C. RNA extraction (Trizol) and qRT-PCR (SYBR Green, β-actin normalization) were performed in triplicate. Lentiviral shRNA constructs targeting RHBDD1/ELK3 (GenePharma) were transfected, with stable clones selected via puromycin and validated by Western blot/qRT-PCR. Proliferation was assessed via CCK-8 (absorbance at 450 nm) and EdU assays (Ribo-Bio kit), while apoptosis was quantified by annexin V-FITC/PI staining using flow cytometry. Immunofluorescence detected β-catenin localization (Abcam antibodies). For *in vivo* analysis, BALB/c nude mice (n = 6/group) received subcutaneous ESCC xenografts, monitored biweekly for tumor volume (L × W^2^/2). IHC evaluated protein expression (Ki67, EMT markers). Data, presented as mean ± SD, were analyzed by Student’s t-test or ANOVA (Dunnett’s *post hoc*; p < 0.05). Protocols followed institutional ethical guidelines.

**Results:**

RHBDD1 promotes cell invasion and migration in ESCC cells. Furthermore, knockdown of RHBDD1 in ESCC cells reduced lung and liver metastasis *in vivo*. The results also indicated that RHBDD1 could promote cell proliferation and inhibit cell apoptosis, which may make ESCC cells more aggressive.

**Conclusion:**

The present study shows that RHBDD1 is an activator of epithelial-mesenchymal transition. This study contributes to the understanding of the role of RHBDD1 in ESCC patients and serves as a valuable resource for in-depth exploration of the pathogenesis of ESCC and the identification of potential therapeutic targets in the future.

## 1 Introduction

Esophageal carcinoma is a highly aggressive malignancy and a significant global health concern. According to the 2018 global cancer statistics, esophageal cancer ranks as the seventh most commonly diagnosed cancer worldwide and the sixth leading cause of cancer-related mortality. This highlights its high prevalence and fatality rate, particularly in East Asian countries where it poses a considerable public health burden (Schweigert et al., 2013; Bray et al., 2024; Global Burden of Disease Cancer et al., 2017; Liu et al., 2023). Notably, in China, esophageal squamous cell carcinoma (ESCC) accounts for the majority of esophageal cancer cases. Various therapeutic strategies, including surgical resection, radiation therapy, and chemotherapy, have been employed in the management of ESCC. More recently, immunotherapy has emerged as a promising treatment modality, offering new hope for patients (Adenis et al., 2022). However, despite advancements in treatment approaches, the high rates of recurrence and metastasis after therapy remain significant obstacles, leading to poor patient prognosis (Domper et al., 2015). Currently, tumor staging, the number of diseased lymph nodes, degree of cell differentiation are often used to evaluate prognostic indicators of esophageal malignant tumors. No specific biomolecular prognostic markers have been widely used in clinical practice yet. Consequently, there is a pressing need to identify key molecular drivers involved in the progression and metastatic potential of ESCC to develop more effective therapeutic interventions.

Among the numerous molecules implicated in cancer pathogenesis, Rhomboid domain containing 1 (RHBDD1) has garnered increasing attention due to its multifaceted role in tumorigenesis. RHBDD1 was initially identified as a highly expressed gene derived from a testicular cDNA library. The first insights into its functional significance emerged in 2008 when studies demonstrated its role in regulating apoptosis in human-derived cells (Wang et al., 2008; Ren et al., 2013). However, it was not until 2013 that RHBDD1 was recognized as a pivotal player in the oncogenesis of various malignancies, including liver, breast, colorectal, and glioma cancers (Liu et al., 2013; Wei et al., 2014; Han et al., 2015; Miao et al., 2017; Zhang M. et al., 2018; Zhang X. et al., 2018; Huang et al., 2018; Wang et al., 2020; Song et al., 2015). Subsequent investigations in 2015 further delineated its mechanistic involvement in colorectal cancer, revealing its ability to cleave proTGF, thereby influencing tumor progression (Song et al., 2015). Additionally, the expression levels of RHBDD1 were found to positively correlate with the activation of the EGFR/Raf/MEK/ERK signaling cascade, a crucial pathway driving colorectal cancer development. Moreover, heightened EGFR expression in colorectal cancer cells has been linked to the activation of the AP-1 transcription factor, a key regulator of cancer proliferation and metastasis (Shen et al., 2018).

Further research in 2018 elucidated the role of RHBDD1 in modulating β-catenin phosphorylation at ser552 and ser675, leading to the activation of the Wnt signaling pathway. This, in turn, upregulated the expression of the Wnt/β-Catenin target gene ZEB1, a transcription factor known to enhance cancer cell migration and invasion, particularly in colorectal cancer (Zhang M. et al., 2018). More recent studies have identified RHBDD1 as a crucial gene targeted by specific microRNAs (MiR-924), further underscoring its significance in cancer progression (Wang et al., 2020). Despite these advancements in understanding RHBDD1’s role in multiple malignancies, its precise function and molecular mechanisms in esophageal cancer remain largely unexplored. Esophageal squamous cell carcinoma (ESCC) remains a significant global health challenge due to its aggressive nature, high recurrence, and metastatic potential, contributing to poor patient prognosis. While Rhomboid domain containing 1 (RHBDD1) has been implicated in the progression of various cancers, its specific role and clinical relevance in ESCC pathogenesis are poorly understood. This study investigated RHBDD1’s function in ESCC. Our findings demonstrate that RHBDD1 acts as a critical activator of epithelial-mesenchymal transition (EMT), significantly promoting ESCC cell proliferation, invasion, migration, and metastasis, while concurrently inhibiting apoptosis. Elucidating RHBDD1’s multifaceted contribution to ESCC aggressiveness provides crucial insights into the disease’s molecular underpinnings. This positions RHBDD1 as a compelling potential therapeutic target, offering a promising avenue for developing novel strategies to combat ESCC progression and metastasis, ultimately aiming to improve patient outcomes.

## 2 Methods

### 2.1 Cell culture

The laboratory cultivated ECA109, a human esophageal squamous cell carcinoma (ESCC) cell line, in RPMI 1640 medium (GIBCO) supplemented with 10% fetal bovine serum (FBS) and antibiotics. To ensure optimal cell growth and viability, all cultures were maintained in a controlled environment within a humidified incubator, set at a constant temperature of 37°C with a 5% CO_2_ atmosphere. Routine monitoring of cell morphology and confluence was conducted using an inverted phase-contrast microscope to assess cell health and adherence. The culture medium was replenished every 48 h to remove metabolic byproducts and maintain an optimal nutrient environment. For sub culturing, cells were enzymatically detached using 0.25% trypsin-EDTA solution (GIBCO), followed by centrifugation at 1,200 rpm for 5 min to isolate the cell pellet. The supernatant was carefully discarded, and the pelleted cells were resuspended in fresh RPMI 1640 medium before being seeded into new flasks or multi-well plates, depending on the specific experimental design. To uphold the integrity of the cell culture, routine *mycoplasma* contamination testing was performed using PCR-based assays, and all experimental procedures were conducted under sterile conditions within a biosafety cabinet to prevent contamination. Cells were consistently maintained in the exponential growth phase prior to any experimental treatments to ensure data reproducibility and accuracy. Additionally, cryopreservation of ECA109 cells was carried out in liquid nitrogen (−196°C) using 10% dimethyl sulfoxide (DMSO) and FBS as cryoprotectants, ensuring long-term viability and genetic stability of the cell line for future studies. Before resuming cultures from cryopreservation, cells were rapidly thawed in a 37°C water bath, washed to remove residual DMSO, and resuspended in fresh medium before being plated. The established cell culture protocol ensured uniformity in experimental conditions, facilitating accurate analyses of esophageal squamous cell carcinoma (ESCC) pathogenesis, molecular mechanisms, and potential therapeutic responses. All experiments were conducted with cells between passages 15 and 25. Cells were routinely monitored for morphology and viability, and no experiments were performed beyond passage 25 to ensure genetic stability.

### 2.2 Extraction and quantitative analysis of RNA using real-time polymerase chain reaction

Total RNA was extracted using Invitrogen’s TRIzol reagent, a widely recognized method for isolating high-quality RNA. The purity and concentration of the extracted RNA were assessed using the NanoDrop ND-1000 spectrophotometer, ensuring accurate quantification and integrity of the samples before further processing. To synthesize complementary DNA (cDNA), reverse transcription (RT) was performed using commercially available kits from Thermo Fisher Scientific and TaKaRa Bio. These kits are specifically designed to enhance the efficiency and accuracy of mRNA reverse transcription, ensuring optimal template preparation for subsequent amplification. Quantitative real-time polymerase chain reaction (qRT-PCR) was conducted using the SYBR Green detection chemistry from Roche. This fluorescent dye-based approach enables precise quantification of target genes by monitoring the amplification process in real-time. The qRT-PCR was performed on a PCR system from Applied Biosystems, a leading platform known for its sensitivity and reproducibility in gene expression studies. To standardize gene expression levels and account for variability in RNA input, β-actin was used as the internal reference gene (housekeeping gene). This normalization approach ensures that any observed changes in gene expression are biologically relevant rather than artifacts of sample processing or technical variations. For specific amplification of CDCA2 (Cell Division Cycle Associated 2), an essential gene involved in cell cycle regulation, a pre-validated All-in-One™ qPCR primer was sourced from GeneCopoeia (Rockville, MD, United States). This primer was designed to optimize specificity and efficiency, minimizing non-specific amplification and enhancing data reliability.

### 2.3 Lentivirus transfection

To establish stable knockdown cell lines, short hairpin RNA (shRNA: Forward sequence (RHBDD1-F): 5′-GCC​TAT​GTT​ATC​ACC​GCA​TTT​TC-3'; Reverse sequence (RHBDD1-R): 5′-GCT​CCT​TTT​GAA​GTC​AGG​TTC​AT-3′) sequences targeting human RHBDD1 and ELK3 were cloned into the hU6-MCS-CMV-Puromycin lentiviral vector (GenePharma, Shanghai, China). Lentivirus particles were produced according to the manufacturer’s instructions and subsequently used to transduce target cells. The transduced cells were subjected to puromycin selection (final concentration: 6 μg/mL) for 7 days to eliminate non-transfected cells and enrich for stable transfectants. pcDNA3.1 vectors containing RHBDD1 and the negative control vectors were purchased from GenePharma. For target gene overexpression, the full-length cDNA was cloned into pcDNA3.1 (+) vector and transfected into cells using Lipofectamine 3,000. Successful transfection was confirmed by Western blot analysis.

### 2.4 Cell viability assay (CCK-8)

Cell proliferation and viability were assessed using the Cell Counting Kit-8 (CCK-8) assay.Cells were seeded at a density of 3,000 cells per well in a 96-well plate, ensuring triplicate wells for each experimental condition. The cells were allowed to adhere overnight before treatment. At specific time points (days 1, 2, 3, and 4), 10 μL of CCK-8 reagent (Dojindo, Japan) was added to each well and incubated for 2 h at 37°C in a humidified incubator with 5% CO_2_. The absorbance of each well was measured at 450 nm using an ELx800 microplate reader (BioTek, United States). The experiment was repeated at least three times, and results were analyzed to compare cell proliferation rates between experimental groups.

### 2.5 Ethynyl deoxyuridine (EdU) incorporation

Cells were analyzed for proliferative ability using a cell Proliferation EdU Image Kit (RiboBio Co., Ltd., Guangzhou, China). 96- well plates were employed to seed cell suspensions, EdU (100 μL 50 μM) was added and incubated for 120 min (46–48 h time window). 4% paraformaldehyde (50 μL) was added for 30 min at room temperature was done for fixing them. Each well was maintained for 5 min with 50 μL 2 mg/mL glycine to neutralize paraformaldehyde. Then, 100 μL 0.5% Triton X-100 was added per well in PBS followed by a 10-min-incubation to enhance cell membrane permeability. DAPI dihydrochloride was applied for 5 min to the samples. Lastly, image acquisition was under a fluorescence microscope.

### 2.6 Apoptosis analysis

Cell apoptosis was evaluated using an Annexin V-FITC/Propidium Iodide (PI) Apoptosis Detection Kit (Vazyme, Nanjing, China). Cells were harvested at 48 h post-treatment, washed with cold PBS, and resuspended in 500 μL of binding buffer. 5 μL of Annexin V-FITC and 5 μL of PI were added to the cell suspension, followed by gentle mixing and incubation for 15 min at room temperature in the dark. Stained cells were analyzed using flow cytometry (FACScan, BD Biosciences, United States), and data were processed using FlowJo software (BD, United States). The percentage of apoptotic cells, including early apoptosis (Annexin V^+^/PI^−^) and late apoptosis/necrosis (Annexin V^+^/PI^+^), was quantified and compared across different experimental groups.

### 2.7 Immunofluorescence staining

Immunofluorescence staining was performed to examine the expression and localization of β-catenin in ESCC cells. Cells were seeded onto glass-bottom dishes, washed twice with PBS, and fixed with 4% paraformaldehyde for 20 min at room temperature. Following fixation, cells were permeabilized with 0.3% Triton X-100 in PBS for 10 min and blocked with 5% bovine serum albumin (BSA) for 1 h to reduce non-specific binding. Cells were then incubated overnight at 4°C with a rabbit anti-β-catenin monoclonal antibody (Abcam, Cambridge, United Kingdom) at a dilution of 1:100. After washing, cells were incubated with an Alexa Fluor 488/555 secondary antibody (Beyotime, Shanghai, China) for 1.5 h at room temperature. Finally, 2 μg/mL DAPI was used to counterstain nuclei for 5 min before imaging with confocal microscopy (Zeiss LSM 880, Germany). Fluorescence intensity and localization of β-catenin were analyzed using ImageJ software (NIH, United States).

### 2.8 Xenograft tumor model in nude mice

All animal experiments were performed in accordance with the guidelines approved by the Institutional Animal Care and Use Committee (IACUC), UCNMU. BALB/c nude mice (4–6 weeks old, female) were purchased from the Animal Center of NMU, China and maintained in a pathogen-free environment with a 12-h light/dark cycle and *ad libitum* access to food and water. Mice were randomly divided into two groups (shCtrl vs shRHBDD1#1, n = 6 per group). Each mouse received a subcutaneous injection of 2 × 10^6^ ESCC cells into the right flank. Tumor growth was monitored every 2 days. Tumor volumes (V) were then computed V=(a × b^2^) × 0.5 (a is length diameter, b is short diameter). At the experimental endpoint, mice were sacrificed, and tumors were excised for further analysis.

### 2.9 Immunohistochemistry

All specimens were fixed in 4% formalin and then embedded in paraffin. Sections (thickness, 4 μm) were incubated overnight at 4°C in primary antibodies with endogenous peroxides and proteins blocked for specific detection of RHBDD1, Ki67, E-cadherin, N-cadherin, and Vimentin (Abcam, Cambridge, United Kingdom). Sections washed by PBS were incubated with HRP‐Polymer‐conjugated secondary antibody at 37°C for 1 h. Subsequently, we stained sections with 3,3‐diaminobenzidine solution for 3 min and counterstained the nuclei were with haematoxylin. The tumour section was examined in a blinded manner. The percentage of positive tumours and the intensity of the cell staining were determined based on three randomly selected regions per section. Staining was scored according to intensity and percentage of positive cells. Tumor tissues were imaged under a Leica DM4 microscope, and staining intensity was quantified using ImageJ software.

### 2.10 Statistical analysis

All experiments were performed at least three times independently, and data are presented as mean ± standard deviation (SD). Statistical significance was set at p < 0.05. Comparisons between two groups were conducted using Student’s t-test, while multiple group comparisons were analyzed using one-way ANOVA, followed by Dunnett’s *post hoc* test for pairwise comparisons. Data analysis was performed using GraphPad Prism 9 (GraphPad Software, United States) and SPSS 26.0 (IBM, United States).

## 3 Results

### 3.1 RHBDD1 promotes cell proliferation of ESCC cells *in vitro*

**FIGURE 1:**
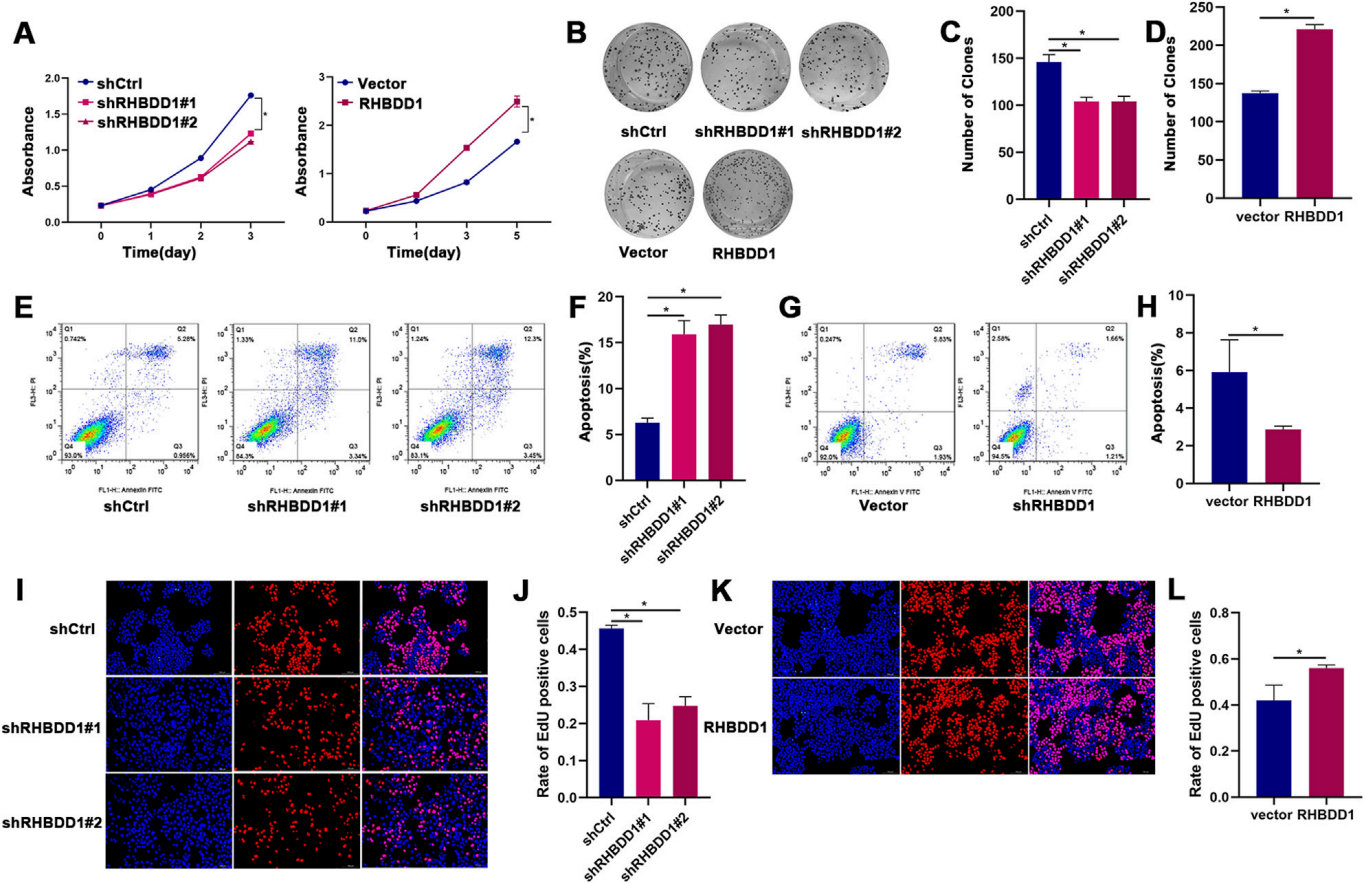
RHBDD1 promotes cell proliferation in esophageal squamous cell carcinoma (ESCC) cells *in vitro*. **(A)** Cell viability assessed by CCK-8 assay:compared with respective control cells. Absorbance values are shown as mean ± SD (n = 3 independent experiments). **(B–D)** Colony formation assays demonstrating RHBDD1’s effect on Eca-109 cells proliferation: **(B)** Representative images of colonies. **(C)** Number of clones of RHBDD1 knockdown group and control group. **(D)** Number of clones of RHBDD1 overexpression group and control group. **(E,F)** The apoptosis of cells between RHBDD1 knockdown group and control group. **(G-H)** The apoptosis of cells RHBDD1 overexpression group and control group. **(I,K)** EdU incorporation assays:Red fluorescence: EdU-positive proliferating cells; Blue fluorescence (DAPI): total nuclei. **(J,L)** Quantification of EdU-positive cells:Proliferation rate calculated as (EdU + cells)/(total cells) × 100%. Data shown as mean ± SD (n = 3 random fields). *p < 0.05 vs. control.

Investigating the effects of RHBDD1 on esophageal cancer, we established cell lines that exhibited either di-minished or amplified expression of RHBDD1. The analysis revealed that the group with reduced RHBDD1 exhibited significantly lower rates of proliferation and colony formation compared to the control group, while their rate of apoptosis was notably higher. On the other hand, the group with enhanced RHBDD1 expression showed opposing results (referenced in Figures 1A–H). Additionally, the influence of RHBDD1 on cell growth was confirmed through EdU incorporation assays, known for their accuracy and selectivity. Eca-109 cells exhibiting decreased RHBDD1 expression showed a reduction in EdU-incorporated cells. Conversely, an enhancement in RHBDD1 ex-pression correlated with a rise in the count of EdU-incorporated cells, as depicted in Figures 1I–L.

### 3.2 RHBDD1 promotes cell migration and invasion of ESCC cells *in vitro*

**FIGURE 2:**
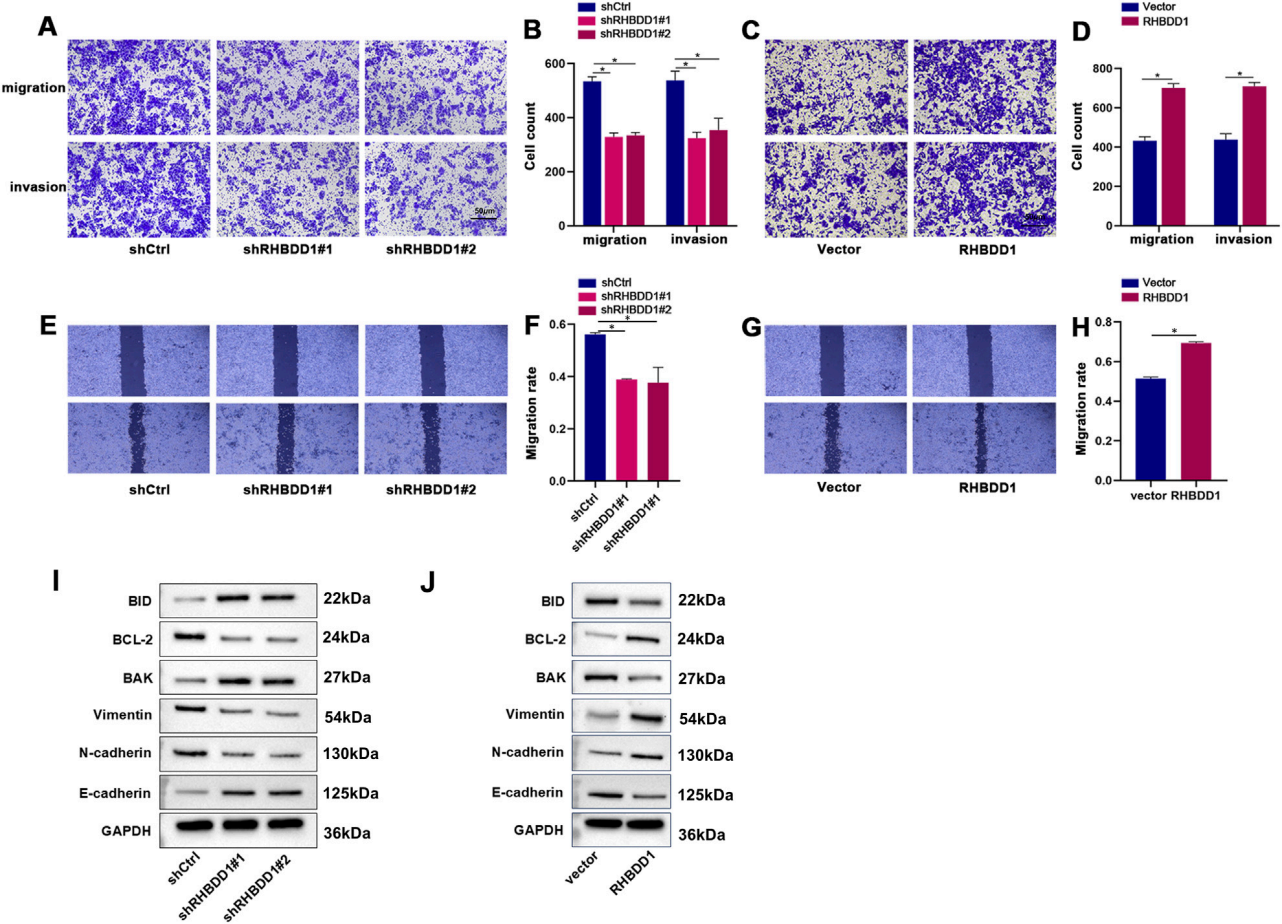
RHBDD1 promotes migration and invasion of esophageal squamous cell carcinoma (ESCC) cells *in vitro* **(A,C)** Representative images of Transwell migration assays in Eca-109 cells compared with respective control cells. **(B,D)** Quantitative analysis of migrated cells from **(A,C)**. **(E,G)** Wound healing assays demonstrating RHBDD1’s effect on Eca-109 cell migration: Images show wound closure at 0 h and 24 h post-scratch. **(F,H)** Quantification of migration rates from wound healing assays, calculated as: (area covered by migrating cells)/(original scratch area) × 100%. **(I,J)** Western blot analysis of epithelial-mesenchymal transition (EMT) and apoptosis-related protein expression:**(I)** EMT markers: Vimentin, E-cadherin, N-cadherin; **(J)** Apoptosis regulators: Bid, Bcl-2, Bak. GAPDH served as internal control protein. (*p < 0.05 vs. respective controls).

Experiments involving wound healing and Transwell assays revealed that reduction of RHBDD1 levels in Eca-109 cells led to a decrease in their invasive and migratory capabilities. In contrast, enhancing RHBDD1 expression in these cells resulted in increased migration rates and a higher number of cells demonstrating invasive behavior *in vitro*, as shown in Figures 2A–H. Additionally, a decrease in RHBDD1 ex-pression was linked to an upregulation of e-cadherin and a downregulation of N-cadherin and Vimentin. Conversely, cells with augmented RHBDD1 expression exhibited the reverse effect, detailed in Figures 2I,J. Additionally, reducing RHBDD1 also led to increased expression of apoptosis-related proteins BAK and BID, alongside a reduction in BCL-2 expression. The inverse was observed in cells with elevated RHBDD1 expression, as indicated in Figures 2I,J.

### 3.3 RHBBD1 regulates the proliferation and metastasis of esophageal cancer cells *in vivo*

**FIGURE 3:**
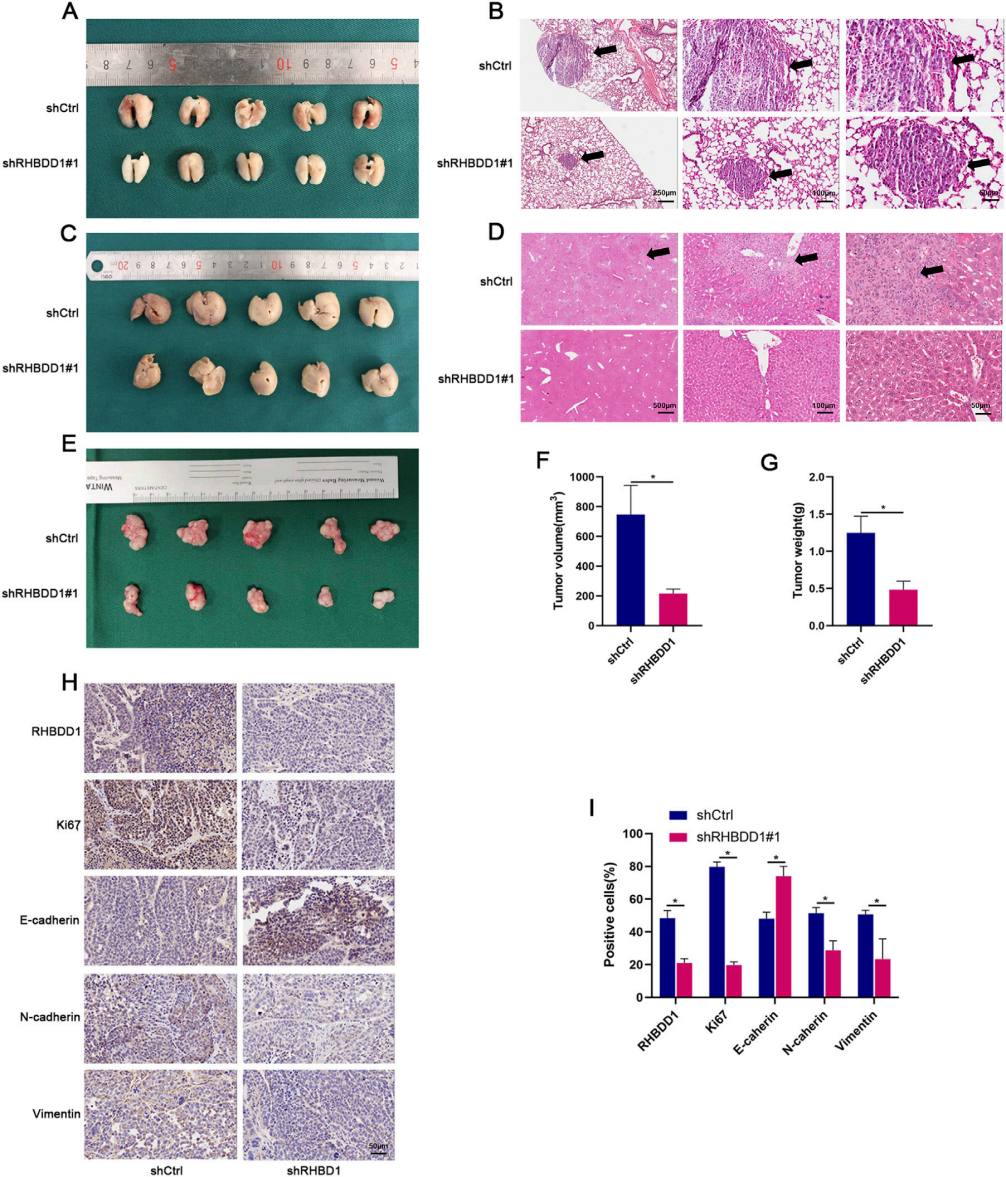
RHBDD1 regulates proliferation and metastasis of esophageal squamous cell carcinoma (ESCC) cells *in vivo*. **(A,C)** Metastatic tumor formation in major organs: **(A)** Representative images of lungs from tail vein injection model. **(C)** Representative images of livers from tail vein injection model. Arrows indicate metastatic nodules. **(E)** tumor growth:Subcutaneous xenograft tumors compared with respective control groups. **(F,G)** Tumor volume quantification:Calculated as V = (a × b^2^) × 0.5, where:a = longest diameter (mm),b = perpendicular diameter (mm). Data presented as mean ± SD (n = 5 mice/group). *p < 0.05 vs. control. **(B,D)** Histopathological analysis:**(B)** H&E staining of lung sections.**(D)** H&E staining of liver sections. Black arrowheads denote metastatic foci. **(H,I)** Immunohistochemical evaluation of RHBDD1, metastasis markers (Vimentin, N-cadherin, E-cadherin) and proliferation marker (Ki67). Positive staining shown in brown (DAB). Scale bars: 50 μm.

In a xenograft mouse model, the influence of RHBDD1 on the proliferation of esophageal cancer was further confirmed. As shown in Supplementary Figure S1, shRHBDD1#1 demonstrated superior knockdown efficiency compared to shRHBDD1#2 in our preliminary *in vitro* validation. Mice injected subcutaneously with RHBDD1 knockdown cells developed significantly smaller tumors compared to the control group. Tumor volume was measured using a digital caliper. The longest diameter (a) and the shortest perpendicular diameter (b) of the tumor were recorded. Tumor volume (V) was calculated using the ellipsoid formula:V=(a × b2) × 0.5. Additionally, mice intravenously injected in the tail with cells expressing RHBDD1 exhibited fewer metastases in the lungs and liver than the control group, as demonstrated in Figures 3A–G. Moreover, tumor tissues from the RHBDD1 knockdown group showed lower levels of Ki67, N-cadherin, and Vimentin, alongside higher expression of E-cadherin, as seen in Figures 3H,I.

### 3.4 RHBDD1 promotes the expression of ELK3 in ESCC cells

**FIGURE 4:**
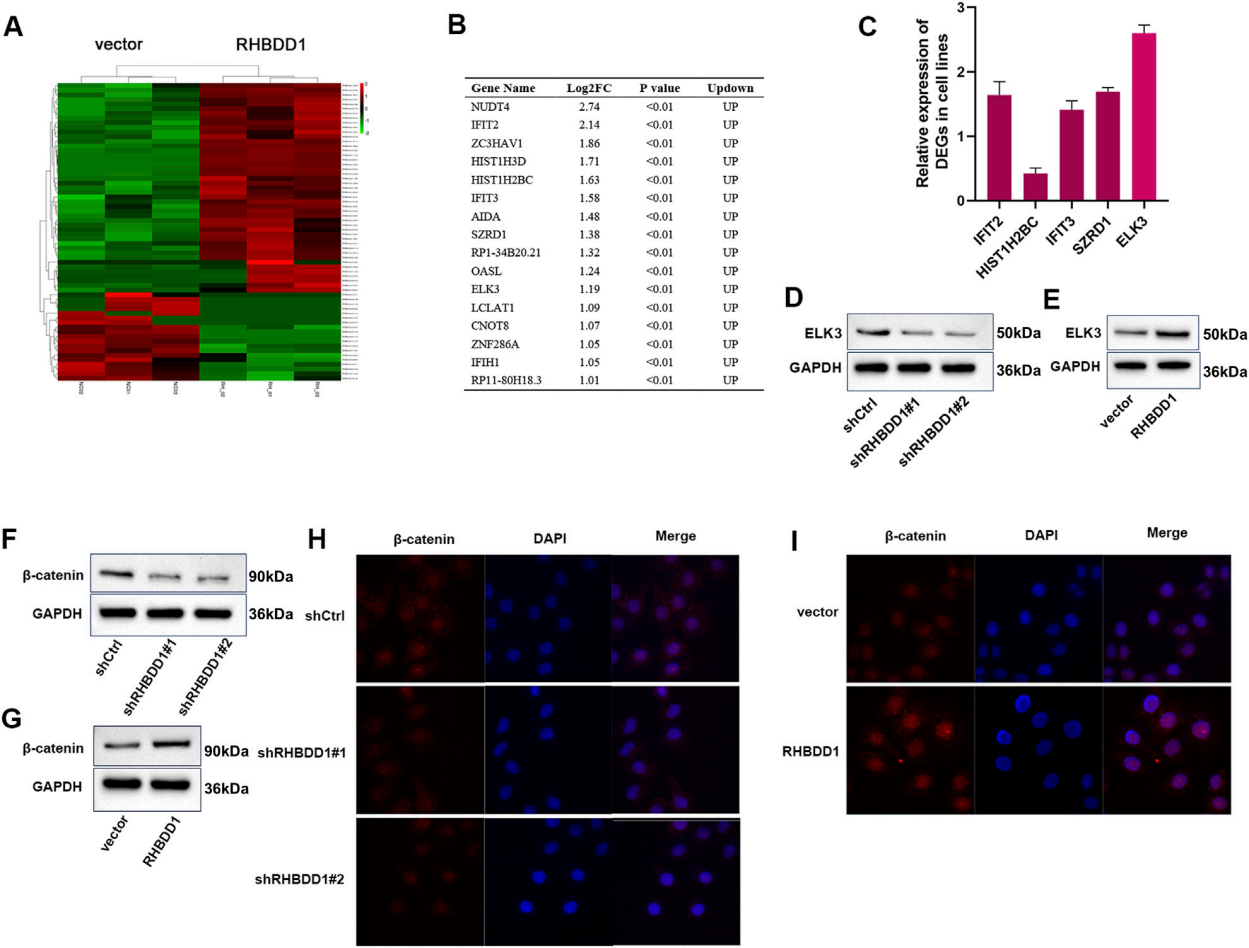
RHBDD1 promotes the expression of ELK3 in Eca-109 cells; **(A)** Heat map of differentially expressed genes between RHBDD1 overexpression cells and Ctrl cells; **(B)** 16 upregulated genes; **(C)** The expressions of IFIT2, HIST1H2BC, IFIT3, SZRD1 and ELK3 in RHBDD1 overexpression and control vector groups cells were detected by qRT-PCR; **(D,E)** Western blot to show the ELK3 expression level; **(F,G)** Western blot to show the β-catenin expression level; **(H,I)** Immunofluorescence plot of nuclear β-catenin expression in esophageal squamous cell carcinoma cells after RHBDD1 modulation.

In our investigation into the mechanistic and molecular networks by which RHBDD1 influences the biological behaviors of ESCC, transcriptome sequencing was conducted on cells from both the RHBDD1 overexpression and control vector groups, with three samples per group. This analysis identified a multitude of differentially ex-pressed genes. Based on criteria including fpkm greater than 1, LogFC greater than 1, and p-value less than 0.05, 16 genes showing upregulation were identified (Figures 4A,B). Among these, five genes previously reported to be involved in tumor development were selected for further study (Shen et al., 2018; Jia et al., 2017; Zeng et al., 2023; Yang et al., 2017; Zhao et al., 2017; Lee et al., 2017). The differential expression of these genes between the RHBDD1 overexpressing and control cell lines was evaluated using qRT-PCR. ELK3 emerged as one of the genes showing significant variation. Previous studies have indicated that ELK3 can facilitate tumor progression, encompassing aspects like invasion and migration. We then verified the disparity in ELK3 expression between the control and RHBDD1 knockdown groups, as well as between the control and RHBDD1 overexpression groups. Significantly, an observed positive relationship was found between the expressions of ELK3 and RHBDD1 in ESCC cells, as illustrated in Figures 4C–E. The association between the Wnt/β-catenin signaling pathway and tumor metastasis has been extensively re-searched, with findings indicating that activation of this pathway can facilitate metastasis. Previous studies have also shown that RHBDD1 in-fluences tumor metastasis via the Wnt/β-catenin signaling pathway (Zhang M. et al., 2018). Our research revealed a direct correlation between the expression of β-catenin and RHBDD1 (Figures 4F, G). Significant alterations in nuclear β-catenin levels were observed in esophageal squamous cell carcinoma cells. In cells where RHBDD1 was knocked down, a substantial reduction in nuclear β-catenin was noted, in contrast to the control group. On the other hand, cells with RHBDD1 overexpressed displayed a notable elevation in nuclear β-catenin levels compared to the control (refer to Figures 4H,I). This data indicates a potential role of RHBDD1 in activating the Wnt/β-catenin signaling pathway in these cells. ELK3 is a well-established oncogenic regulator. Given that miR-135a targets ELK3’s 3′UTR in breast cancer (Ahmad et al., 2018), we propose investigating whether RHBDD1 transcriptionally regulates ELK3 using luciferase reporter assays. This approach would determine if RHBDD1 modulates ELK3 promoter activity or mRNA stability, providing mechanistic insights into their functional relationship.

### 3.5 Konckdown of ELK3 expression in esophageal squamous cell carcinoma can reverse the invasion and migration promoted by RHBDD1

**FIGURE 5:**
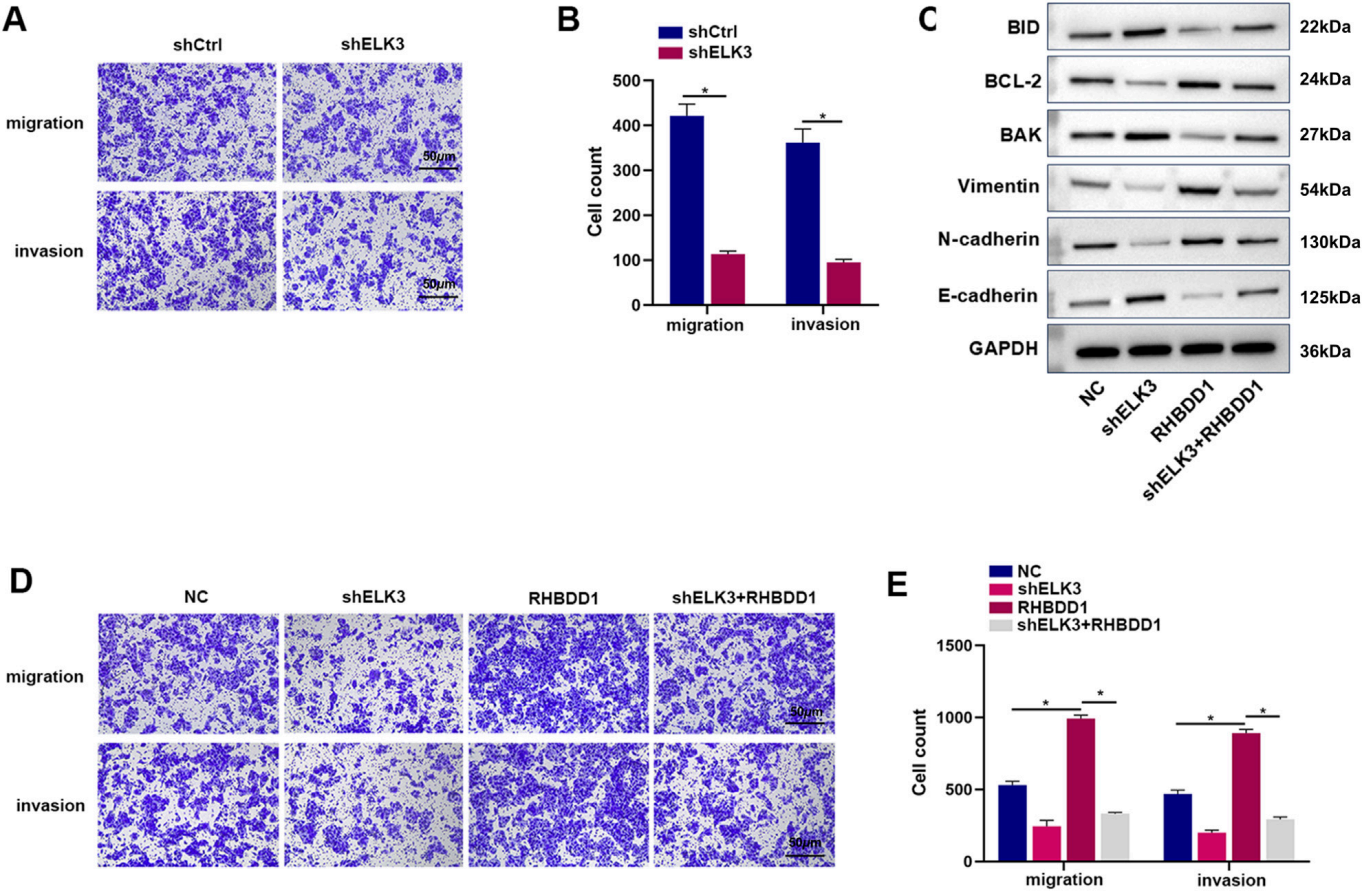
Inhibition of ELK3 expression in esophageal squamous cell carcinoma can reverse the invasion and migration pro-moted by RHBDD1; **(A,B)** After the downregulation of ELK3, the invasion and migration of Eca-109 cells decreased. The histogram depicts the quantification of the migrated cells. **(C)** Western blot to show the EMT-related and apoptosis-related proteins expression levels; **(D,E)** ELK3 could partially reverse the RHBDD1-induced increase in invasion and migration of Eca-109 cells. The histogram depicts the quantification of the migrated cells. (*means p < 0.05).

Transwell experiments revealed that diminishing ELK3 in Eca109 cells led to a decrease in their invasion and migration capabilities (Figures 5A, B). To determine if ELK3’s regulation of the invasion and migration abilities in esophageal squamous cell carcinoma cells is linked to RHBDD1, we performed experiments with overexpressed RHBDD1 and downregulated ELK3. Results from the Transwell assays indicated that the group with RHBDD1 overexpression exhibited a significant enhancement in invasion and migration abilities compared to the control group. However, in the group where RHBDD1 was overexpressed and ELK3 was knocked down, these abilities were reduced, aligning the cell numbers more closely with those of the control group (Figures 5D, E). Western blotting was used to compare epithelial-mesenchymal markers (including Ecadherin and N-cadherin) between the control group, the ELK3 knockdown group, the RHBDD1 overexpression group, and the RHBDD1 overexpression + ELK3 knockdown group. It was found that compared with the control group, E-cadherin in the ELK3 downregulation group was significantly increased, while the expression levels of N-cadherin and vimentin decreased. This suggests that down-regulating the expression level of ELK3 in esophageal squamous cell carcinoma inhibits the epithelial-mesenchymal transition process to a certain extent. Correspondingly, compared with the RHBDD1 overexpression group, the expression of E-cadherin in the RHBDD1 overexpression + ELK3 downregulation group decreased, while the expressions of N-cadherin and vimentin increased. In addition, similar results were obtained for the expression of apoptosis-related proteins (Figure 5C). These findings, in conjunction with the previously verified positive correlation between ELK3 and RHBDD1 in Eca-109 cells, suggest that the knockdown of ELK3 can counteract the RHBDD1-induced promotion of invasion and migration in these cells.

## 4 Discussion

RHBDD1 expression was significantly elevated in ESCC cells. Functionally, RHBDD1 was demonstrated to promote the invasion and migration capabilities of ESCC cells *in vitro*. Critically, *in vivo* studies using nude mouse xenograft models showed that knocking down RHBDD1 expression in ESCC cells significantly reduced metastasis to the lungs and liver. Further mechanistic investigations revealed that RHBDD1 contributes to ESCC progression by enhancing cell proliferation (as measured by CCK-8 and EdU assays) and simultaneously inhibiting apoptosis (quantified by annexin V-FITC/PI flow cytometry). This dual effect of RHBDD1, stimulating growth while preventing programmed cell death, suggests it plays a role in making ESCC cells more aggressive. Collectively, these results establish RHBDD1 as a potential driver of proliferation, survival, invasion, migration, and metastasis in esophageal squamous cell carcinoma.

While this study provides mechanistic insights into the role of RHBDD1 in regulating the ELK3-Wnt/β-catenin axis in ESCC, we acknowledge that our findings are primarily derived from experiments using the ECA109 cell line. Given the known heterogeneity of ESCC and potential concerns regarding cell line cross-contamination, future studies will validate these results in additional, well-characterized ESCC models (e.g., KYSE-150, TE-1) with rigorous authentication. This will further strengthen the translational relevance of our conclusions.

Esophageal cancer, particularly esophageal squamous cell carcinoma, ranks highly in terms of incidence and mortality within the spectrum of cancers in East Asia. This carcinoma type predominantly impacts the swallowing function in advanced stages, leading to poor nutritional status and significant pain for most patients. Hence, identifying new potential targets for this carcinoma is crucial for reducing the overall burden of esophageal cancer and alleviating patient suffering. Tumor metastasis, a complex multistep process, involves the spread of tumor cells from their origin to distant sites. The process encompasses several stages, such as invasion into adjacent tissues, migration through blood vessels, infiltration into remote locations, and subsequent colonization and proliferation (Gao et al., 2019; Strilic and Offermanns, 2017). At diagnosis, a majority of patients already exhibit invasion into the muscular layer, and approximately 50% have tumors that have metastasized to nearby organs, lymph nodes, liver, and lungs (Gu et al., 2012).

In this study, we observed that RHBDD1 is notably upregulated in ESCC cells, affirming its role in enhancing cell invasion and migration. Additionally, RHBDD1 knockdown in ESCC cells led to reduced metastasis to lungs and liver *in vivo*. Previous studies have also indicated RHBDD1’s role in promoting invasion and migration in cancers such as rectal cancer, NSCLC, and breast cancer. Our findings suggest that RHBDD1 contributes to increased cell proliferation and reduced apoptosis, potentially heightening the aggressiveness of ESCC cells. EMT, a critical factor in metastasis, is influenced at various levels by different EMT activators (Brabletz et al., 2001; Vincent et al., 2009; Kahlert et al., 2011; Paterson et al., 2013). Our research identifies RHBDD1 as an activator of EMT.

In our study, it was observed that RHBDD1 levels were markedly elevated in ESCC cells, promoting enhanced cell invasion and migration. Furthermore, the reduction of RHBDD1 in these cells led to decreased metastasis to the lungs and liver. Prior research has indicated that RHBDD1 also facilitates invasion and migration in various cancers, including rectal cancer, NSCLC, and breast cancer. In ESCC cells, elevated levels of RHBDD1 were correlated with enhanced cell proliferation and diminished apoptotic activity, leading to a more malignant cancer profile. While RHBDD1 promotes colorectal cancer (CRC) via TGFα/EGFR/ERK signaling (Song et al., 2015), our study reveals a distinct mechanism in ESCC, where RHBDD1 enhances invasion by activating Wnt/β-catenin via ELK3. This contrast highlights RHBDD1’s context-dependent roles—driving proliferation in CRC and metastasis in ESCC—suggesting tissue-specific therapeutic targeting strategies. The process of EMT, crucial in cancer cell transformation and metastasis promotion, is regulated by several factors, includ-ing RHBDD1 (Singh et al., 2018). To delve deeper into RHBDD1’s impact on the growth of ESCC cells, transcriptome sequencing analysis was performed. This revealed a positive correlation between the expressions of ELK3 and RHBDD1, con-firmed at both the RNA and protein levels. Previous research has shown ELK3’s association with tumor invasion and migration. Notably, ELK3, as a target gene of microRNA in triple-negative breast cancer, plays a regulatory role in metastasis (Kim et al., 2020). Enhanced expression of RHBDD1 in ESCC cells significantly increased tumor cell invasion and migration. Conversely, the downregulation of ELK3 in these cells led to a significant inhibition of these processes compared to the control group. However, the cell invasion and migration enhancement induced by RHBDD1 upregulation were mitigated when ELK3 expression was further reduced. This study also investigated the Wnt/β-catenin signaling pathway’s connection with tumor invasion and migration. We found that β-catenin ex-pression was inhibited when RHBDD1 was downregulated and increased when RHBDD1 was upregulated. Immunofluorescence assays revealed alterations in RHBDD1 levels impacting the expression of β-catenin within the nucleus, suggesting a potential role of RHBDD1 in activating the Wnt/β-catenin signaling pathway, thereby facilitating invasion and migration of ESCC cells. These findings suggest that RHBDD1 may serve as a novel promoter of tumorigenesis in ESCC, promoting tumor metastasis and proliferation while suppressing apoptosis. Through the regulation of ELK3 and facilitation of EMT and Wnt/β-catenin pathway activation, RHBDD1 holds promise as a potential therapeutic target for ESCC treatment. While our study identified ELK3 as a key RHBDD1 downstream effector, future work will: validate alternative candidates. Elucidate ELK3/β-catenin mechanistic links via rescue and interaction assays, and establish translational relevance using xenograft models.

The development of RHBDD1-targeted therapies faces significant challenges, including potential off-target effects due to structural homology with other rhomboid proteases (e.g., PARL, RHBDL2), which may disrupt mitochondrial or growth factor signaling. To enhance specificity, strategies such as allosteric inhibition or proteolysis-targeting chimeras could exploit unique conformational epitopes of RHBDD1. Additionally, poor bioavailability and *in vivo* instability of inhibitors necessitate advanced delivery systems—lipid nanoparticles or antibody-drug conjugates may improve tumor-selective accumulation. Biomarker-driven approaches, such as screening tumors for RHBDD1/EGFR or Wnt/β-catenin pathway activation, could further refine patient stratification. Overcoming these hurdles will require combinatorial innovations in drug design and precision medicine to translate RHBDD1 inhibition into clinical success.

## 5 Conclusion

In this study, we found t RHBDD1 promotes cell invasion and migration in ESCC cells. Furthermore, knockdown of RHBDD1 in ESCC cells reduced lung and liver metastasis *in vivo*. Our results also indicated that RHBDD1 could promote cell proliferation and inhibit cell apoptosis, which may make ESCC cells more aggressive. In the present study, we show that RHBDD1 is an activator of epithelial-mesenchymal transition. We believe that our study will contribute to the understanding of the role of RHBDD1 in ESCC patients and serve as a valuable resource for in-depth exploration of the pathogenesis of ESCC and the identification of potential therapeutic targets in the future.

## Data Availability

The original contributions presented in the study are publicly available. This data can be found here: https://doi.org/10.6084/m9.figshare.29618456.v1.
